# Attachment Avoidance Moderates the Relationship Among Acceptance of Sugar Relationships, Motivation, and Self-Esteem

**DOI:** 10.3389/fpsyg.2021.711199

**Published:** 2021-08-16

**Authors:** Dóra Ipolyi, Edit Csányi, András Láng, Norbert Meskó

**Affiliations:** Institute of Psychology, University of Pécs, Pécs, Hungary

**Keywords:** acceptance of sugar relationships, adult attachment, self-esteem, motivation, transactional sex

## Abstract

A sugar relationship is a monetary-based liaison between a wealthier older and an attractive younger person, the latter receiving expensive gifts or financial compensation in exchange for her or his sexual companionship. Attachment, motivation, and self-esteem are all integrative parts of adult romantic and sexual relationships. There is relatively little empirical research on the psychological correlates of accepting attitudes toward sugar relationships. The research aimed to explore the relationship among the acceptance of sugar relationship, attachment (avoidance and anxiety), motivation (extrinsic and intrinsic), and self-esteem. A total of 2,409 Hungarian adults including 1,980 younger participants (1,804 women, 175 men, mean age = 21.17) and 429 older participants (290 men, 138 women, mean age = 48.86) completed an online test battery comprising four self-report measures. In the younger subsample, the only significant association obtained for the acceptance of sugar relationships was its positive correlation with extrinsic motivation. In the older subsample, the acceptance of sugar relationships was positively correlated with all tested variables except self-esteem. Subsequent analysis revealed that attachment avoidance but not anxiety moderated the associations between the variables. Among younger participants, the negative effect of self-esteem and intrinsic motivation on accepting sugar relationships decreased with increasing attachment avoidance. Among older participants, the positive effect of extrinsic motivation on accepting sugar relationships decreased with increasing attachment avoidance. The results are discussed with regard to relational and sexual goals associated with adult attachment orientations.

## Theoretical Background

### Sugar Relationship

Transactional sex includes specific forms of sexual relationship based on an exchange of sexual companionship and material support or other benefits. A sugar relationship is a modern form of transactional sexual relationship, in which a younger partner (sugar baby/boy) receives material compensation from an older and wealthier partner (sugar daddy/mommy) in return for her or his companionship (Nayar, 2016). The majority of studies on the psychology of sugar relationships focus on the younger partners (e.g., Hoss and Blokland, 2018) in terms of their personality traits (e.g., Blum et al., 2018) and the support provided by their parents (e.g., Betzer et al., 2015). In a recent study, the acceptance attitude of young participants toward engaging in sugar relationships was positively related to a game-playing love style, egoistic sexual motivation, a more unrestricted sociosexual orientation, and socially aversive personality traits, such as Machiavellianism, subclinical psychopathy, and borderline personality organization (Birkás et al., 2020). Much fewer studies focus on the older (and wealthier) partners in sugar relationships, partly due to difficulties with sampling individuals currently or previously involved in a sugar relationship (Betzer et al., 2015). Recently, Láng et al. (2021) developed an attitude scale to assess the acceptance of sugar relationships by older women and men. The authors found that higher acceptance of sugar relationships was associated with more unrestricted sociosexuality, higher preference for engaging in playful love relationships, higher self-focused sexual motivation, and more pronounced Dark Triad, and borderline traits. Both Birkás et al. (2020) and Láng et al. (2021) revealed that older adults reporting an accepting attitude toward sugar relationships had sexual motives and personality traits similar to those of younger adults who reported willingness to engage in transactional sex.

### Motivation

Aspirations are long-term goals manifested in behaviors varying across individuals and situations (Kasser and Ryan, 1993,1996). These goals and the underlying motives have a dual nature. Intrinsically motivated aspirations serve the psychological needs of oneself (e.g., self-acceptance, personal development, social relationships, a sense of communion, and physical health), while extrinsically motivated aspirations are aimed at rewards provided by the external environment (e.g., financial and social success, reputation, and physical attractiveness; Kim et al., 2003).

Intrinsic motivation is more closely associated with general well-being, satisfaction with life, happiness, and balanced sex life, whereas extrinsic motivation is primarily related to immediate rewards, tangible benefits, and short-term need satisfaction. Extrinsic motivation shows a negative or no association with well-being, while it is positively related to low self-esteem, addictions, and poor social and sexual relationships (Rijavec et al., 2006). External regulation of sex life is the least autonomous type of extrinsic motivation, where one engages in sexual activity to avoid conflict or punishment or to gain benefits. Externally regulated sexual behavior is not consistent with one's self. On contrary, intrinsic regulation is the most autonomous type of sexual motivation, in which case the actions of an individual are fully consistent with the will of an individual (Gravel et al., 2016).

The acceptance of sugar relationships showed a positive relationship with Machiavellianism to both younger (Birkás et al., 2020) and older adults (Láng et al., 2021). Since, as McHoskey (1999) points out, Machiavellians are primarily driven by external rewards (e.g., status, resources), rather than intrinsic motives (e.g., community, family, and romantic love), the acceptance of sugar relationships is likely to be related to the extrinsic-intrinsic dimensions of motivation.

### Attachment

According to Hazan and Shaver (1987), early infant-caregiver attachment forms the basis of attachment patterns in intimate partner relationships of adults, in which the partner replaces the caregiver as the attachment figure. Both infant and adult attachment shows three typical patterns including the secure, anxious, and avoidant styles (Bartholomew and Horowitz, 1991); however, while infant attachment to the caregiver is an asymmetrical, dependency-based, and non-sexual relationship, adult attachment, normally, is symmetrical and reciprocal, and it involves sexuality (Hazan and Zeifman, 1994).

In intimate partner relationships, the secure vs. insecure (anxious or avoidant) attachment style of an individual has a decisive impact on attitudes of an individual toward that partner and characteristic modes of an individual of coping with relationship problems. Securely attached adults showed relatively low levels of anxiety and avoidance, which were associated with supportive and accepting attitudes toward their partners and with high levels of relationship satisfaction, whereas, insecurely attached adults had less positive social experiences and more difficulties with approaching others, and they were less satisfied with their intimate partner relationships (Vicary and Fraley, 2009). Those with an anxious attachment style were prone to engage in casual sexual encounters lacking intimate interactions and adequate need for fulfillment, while those characterized by avoidance attachment tended to engage in emotionally detached sexual relationships (Hazan and Shaver, 1987). Potard et al. (2014) found that women who had avoidance attachment with their fathers during childhood had difficulties with sexual choices in adulthood. On the contrary, their securely attached counterparts had fewer, more emotionally balanced, intimate, and satisfying sexual relationships (Birnbaum et al., 2006).

Fraley and Shaver (2000) suggest that individuals regulate their attachment behavior by setting a subjectively optimal level of avoidance, that is, emotional distance from the attachment figure, whose discrepancy from the currently perceived distance elicits anxiety in them.

### Self-Esteem

Self-esteem is the evaluative component of the self-concept, which is reflected in feelings about important things of an individual, and treatment of oneself (Rosenberg et al., 1995). Self-esteem may also be defined as a type of sociometer, which enables one to appraise the subjective value of members of an important or desirable group (Sowislo and Orth, 2012). Several theoretical models propose that self-esteem is a pervasive human motive, which generally serves adaptive behavior and contributes to favorable outcomes for the individual (Pyszczynski et al., 2004). This approach has led to the pragmatic assumption that people are motivated to maintain high self-esteem. As Baumeister et al. (2003) point out, however, subjective experiences may result in fluctuations of self-esteem: achieving significant success (e.g., winning in a competition, solving a problem, attaining a goal) seems to be associated with increased state self-esteem, while failures are related to temporary decreases in self-esteem. Low self-esteem emerging in adolescence is a risk factor for subsequent mental and physical health conditions, poor financial prospects, and criminal conduct (Trzesniewski et al., 2006), and for an early start of sex life, high-risk sexual activity, and a large number of sexual partners (Ethier et al., 2006).

## Aims of Research

In consistence with the theoretical background discussed, the acceptance of sugar relationships was expected to be positively associated with extrinsic rather than intrinsic motivation and with avoidance rather than an anxious attachment. In the absence of relevant previous findings, no specific prediction was made for its association with self-esteem. To the best of our knowledge, the present study is the first to explore the psychological correlations of the acceptance of sugar relationships.

## Method

### Sample and Procedure

The overall sample (*N* = 2,409) comprised a younger and an older subsample (*n* = 1,980 and 429, respectively). The younger subsample included 175 males and 1,804 females (one participant did not indicate their gender) aged between 18 to 27 years (*M* = 21.17 and *SD* = 2.91). The older subsample included 290 men and 138 women (one participant did not indicate their gender) aged between 40 to 71 years (*M* = 48.86 and *SD* = 7.33).

Data were collected online. The survey was edited in Google forms. The link to the survey was disseminated *via* Facebook and one of the most popular and influential Hungarian Internet portals, Index (https://index.hu). All participants gave their written informed consent, and none of them was rewarded for participation. The research plan received ethical approval from the Hungarian United Ethical Review Committee for Research in Psychology (Ref. No. 2018/115).

### Measures

#### The Acceptance of Sugar Relationships in Young Women and Men Scale (ASR-YWMS)

The ASR-YWMS (Birkás et al., 2020) comprises five items assessing the willingness in oneself to engage in sugar relationships as the younger partner (potential sugar baby/boy) provides her/his sexual companionship for the older partner in return for gifts and/or monetary compensation. The participants rated each item on a 5-point scale ranging from 1 indicating extreme rejection (e.g., absolutely disagree) to 5 indicating extreme acceptance (e.g., absolutely agree). In the present study, the ASR-YWMS showed adequate internal consistency (Cronbach's α = 0.940).

#### The Acceptance of Sugar Relationships in Older Men and Women Scale (ASR-OMWS)

The ASR-OMWS (Láng et al., 2021) comprises five items assessing the willingness in oneself to engage in sugar relationships as the older partner (potential sugar daddy/mommy) provides gifts and/or monetary compensation for the younger partner in return for her/his sexual companionship. The participants rated each item on a 5-point scale ranging from 1 indicating extreme rejection (e.g., *absolutely disagree*) to 5 indicating extreme acceptance (e.g., *absolutely agree*). The scale showed adequate internal consistency (Cronbach's α = 0.939).

#### Relationship Structures of Experiences in Close Relationships (ECR-RS)

The ECR-RS scale (Fraley et al., 2011; Hungarian adaptation by Jantek and Vargha, 2016) assesses anxious and avoidance attachment styles with a total of nine items concerning the close relationships with the respondent in general. Participants rated each item on a 7-point scale ranging from *strongly disagree* (1) to *strongly agree* (7). A mean score was computed for each of the avoidance and anxiety subscales. Higher scores on each subscale indicated higher attachment insecurity. Both subscales showed adequate internal consistency (α = 0.864 and 0.892, respectively).

#### Rosenberg Self-Esteem Scale (RSES)

The Hungarian version of the 10-item RSES-H (RSES, Rosenberg, 1965; Hungarian adaptation by Sallay et al., 2014) provides a measure of global self-esteem. The participants rated their agreement with each item on a 4-point scale ranging from *strongly disagree* (0) to *strongly agree* (3). Higher scores indicated higher global self-esteem. The scale showed adequate internal consistency (Cronbach's α = 0.920).

#### Aspiration Index

The AI (Kasser and Ryan, 1996; Hungarian adaptation by Martos et al., 2006) assesses personal aspirations defined as long-term overall goals, which indicate the basic direction of the life of the respondent. The Likert scale comprises two subscales measuring intrinsically motivated aspirations (e.g., meaningful social relationships, personal growth, and commitment to the community) and extrinsically motivated aspirations (e.g., wealth, reputation, and attractive appearance). Both intrinsic aspirations and extrinsic aspirations showed adequate internal consistency (α = 0.722 and 0.823, respectively).

## Results

We used Pearson's correlations to test the associations between measured variables. The results are presented for both age groups separately. For younger participants (Table 1), the acceptance of sugar relationships showed significant correlations with all measured variables; however, the strengths of correlations were negligible (i.e., *r* < |0.2|; Ferguson, 2009) except for extrinsic motivation (*r* = 0.279; *p* < 0.001). A more accepting attitude toward sugar relationships was associated with more intense extrinsic motivation.

**Table 1:** Associations of measured variables with the acceptance of sugar relationships; the results of Pearson's correlations for the younger subsample (*n* = 1,980).

	**ASR-YWMS**	**ECR-RS AV**	**ECR-RS AX**	**RSES**	**AI-I**
ECR-RS AV	0.151^***^				
ECR-RS AX	0.179^***^	0.289^***^			
RSES	−0.082^***^	−0.281^***^	−0.510^***^		
AI-I	−0.171^***^	−0.293^***^	0.017	0.050^*^	
AI-E	0.279^***^	−0.046^*^	0.128^***^	0.056^*^	0.145^***^

* *p < 0.05*;

** *p < 0.01*;

*** *p < 0.001*.

*ASR-YWMS, Acceptance of Sugar Relationships in Young Women and Men Scale; ECR-RS AV, Experiences in Close Relationships-Relationship Structures Scale attachment avoidance dimension; ECR-RS AX, Experiences in Close Relationships-Relationship Structures Scale Attachment Anxiety Dimension; RSES, Rosenberg Self-Esteem Scale; AI-I, Aspirations Index-Intrinsic; AI-E, Aspirations Index-Extrinsic*.

For older participants (Table 2), the acceptance of sugar relationships showed significant associations with all measured variables except for self-esteem (*r* = −0.045; *p* = 0.356). Attachment avoidance (*r* = 0.300; *p* < 0.001), attachment anxiety (*r* = 0.252; *p* < 0.001), and extrinsic motivation (*r* = 0.302; *p* < 0.001) showed positive correlations with the acceptance of sugar relationships with weak to moderate strength. Intrinsic motivation was negatively correlated with the acceptance of sugar relationships with weak strength (*r* = −0.270; *p* < 0.001). Thus, a more accepting attitude toward sugar relationships was associated with higher fear of both intimacy and separation, more intense extrinsic and less intense intrinsic motivations.

**Table 2:** Associations of measured variables; the results of Pearson's correlations for the older subsample (*n* = 429).

	**ASR-OMWS**	**ECR-RS AV**	**ECR-RS AX**	**RSES**	**AI-I**
ECR-RS AV	0.300^***^				
ECR-RS AX	0.252^***^	0.293^***^			
RSES	−0.045	−0.223^***^	−0.435^***^		
AI-I	−0.270^***^	−0.346^***^	−0.170^***^	0.261^***^	
AI-E	0.302^***^	0.021	0.110^*^	0.044	0.099^*^

* *p < 0.05*;

** *p < 0.01*;

*** *p < 0.001*.

*ASR-OMWS, Acceptance of Sugar Relationships in Older Men and Women Scale; ECR-RS AV, Experiences in Close Relationships-Relationship Structures Scale attachment avoidance dimension; ECR-RS AX, Experiences in Close Relationships Structures Scale Attachment Anxiety Dimension; RSES, Rosenberg Self-Esteem Scale; AI-I, Aspirations Index-Intrinsic; AI-E, Aspirations Index-Extrinsic*.

Next, we tested the moderation effect of attachment anxiety and attachment avoidance on the relationship between self-esteem, motivation (both intrinsic and extrinsic), and the acceptance of sugar relationships. Moderation analyses were run with PROCESS 3.1. (Hayes, 2018). A total of 12 moderation analyses were run (6–6 for both subsamples). The results are summarized in Tables 3, 4. For the younger subsample (Table 3), attachment anxiety had no significant moderation effect. Attachment avoidance moderated the self-esteem to the effect of the acceptance of sugar relationships. Estimated effects of self-esteem on the acceptance of sugar relationships were −0.13 (*p* < 0.001), −0.07 (*p* = 0.017), and 0.02 (*p* = 0.541) at 1 SD below mean, mean, and 1 SD above mean values of attachment avoidance. Thus, with the increase in attachment avoidance, the negative effect of self-esteem on the acceptance of sugar relationships waned and disappeared. Attachment avoidance also moderated the intrinsic motivation to the acceptance of sugar relationships effect. Estimated effects of intrinsic motivation on the acceptance of sugar relationships were −0.28 (*p* = 0.013), −0.42 (*p* < 0.001), and −0.60 (*p* < 0.001) at 1 SD below mean, mean, and 1 SD above mean values of attachment avoidance. Thus, with the increase in attachment avoidance, the negative effect of intrinsic motivation on the acceptance of sugar relationships increased in strength.

**Table 3:** Mediation effects of attachment avoidance and attachment anxiety on the self-esteem/motivation to the effect of the acceptance of sugar relationships in younger participants (*n* = 1,980); unstandardized coefficients.

**Independent variable (X)**	**X**	**M**	**X × M interaction**
**Mediator (M)** **=** **ECR-RS AV**			
RSES	−0.217^***^	−0.116	0.011^**^
AI-I	−0.098	0.752^**^	−0.023^*^
AI-E	0.549^***^	0.156	0.002
**Mediator (M)** **=** **ECR-RS AX**			
RSES	−0.045	0.135	0.005
AI-I	−0.398^*^	0.816^*^	−0.020
AI-E	0.398^***^	−0.020	0.012

* *p < 0.05*;

** *p < 0.01*;

*** *p < 0.001*.

*ASR-YWMS, Acceptance of Sugar Relationships in Young Women and Men Scale is the dependent variable in all cases. ECR-RS AV, Experiences in Close Relationships-Relationship Structures Scale Attachment Avoidance Dimension; ECR-RS AX, Experiences in Close Relationships-Relationship Structures Scale Attachment Anxiety Dimension; RSES, Rosenberg Self-Esteem Scale; AI-I, Aspirations Index-Intrinsic; AI-E, Aspirations Index-Extrinsic*.

**Table 4:** Mediation effects of attachment avoidance and attachment anxiety on the self-esteem/motivation to effect of the acceptance of sugar relationships in older participants (*n* = 429); unstandardized coefficients.

**Independent variable (X)**	**X**	**M**	**X × M interaction**
**Mediator (M)** **=** **ECR-RS AV**			
RSES	−0.089	0.163	0.007
AI-I	−1.055^**^	−0.243	0.021
AI-E	1.312^***^	0.922^***^	−0.294^*^
**Mediator (M)** **=** **ECR-RS AX**			
RSES	0.134	0.564	0.002
AI-I	−1.152^***^	−0.631	0.043
AI-E	1.081^***^	1.256^**^	−0.042

* *p < 0.05*;

** *p < 0.01*;

*** *p < 0.001*.

*ASR-OWMS, Acceptance of Sugar Relationships in Older Men and Women Scale is the dependent variable in all cases. ECR-RS AV, Experiences in Close Relationships-Relationship Structures Scale Attachment Avoidance Dimension; ECR-RS AX, Experiences in Close Relationships-Relationship Structures Scale Attachment Anxiety Dimension; RSES, Rosenberg Self-Esteem Scale; AI-I, Aspirations Index-Intrinsic; AI-E, Aspirations Index-Extrinsic*.

For the older subsample (Table 4), only one significant moderation effect was found. Attachment avoidance moderated the extrinsic motivation to the acceptance of sugar relationships effect. Estimated effects of extrinsic motivation on the acceptance of sugar relationships were 1.05 (*p* < 0.001), 0.81 (*p* < 0.001), and 0.55 (*p* < 0.001) at 1 SD below mean, mean, and 1 SD above mean values of attachment avoidance. Thus, with the increase in attachment avoidance, the positive effect of extrinsic motivation on the acceptance of sugar relationships decreased in strength.

## Discussion

The present study explored the psychological correlates of attitudes of younger (potential sugar babies/boys) and older individuals (potential sugar daddies/mommies) toward sugar relationships. To the best of our knowledge, this study is the first to have explored the associations of the acceptance of sugar relationships with extrinsic and intrinsic motivation, self-esteem, and attachment anxiety and avoidance.

In the younger subsample, the acceptance of sugar relationships only showed an association of considerable magnitude with extrinsic motivation. In the older subsample, by contrast, the acceptance of sugar relationships was meaningfully associated with all tested variables except self-esteem. Positive correlations were found for attachment avoidance, attachment anxiety, and extrinsic motivation, while intrinsic motivation was negatively correlated with the acceptance of sugar relationships. Overall, higher acceptance of sugar relationships was associated with a more intense fear of both intimacy and separation, higher levels of extrinsic motivation, and lower levels of intrinsic motivation. These results are in part consistent with related previous findings revealing positive associations of insecure attachment with pretended orgasm reported by women (Láng et al., 2020), vibrator use, anal sex (Costa and Brody, 2011), and sexual addictions in men (Zapf et al., 2008).

The results of the moderation analyses showed that attachment anxiety of younger participants did not have a significant moderating effect on their self-esteem, intrinsic motivation, extrinsic motivation, or the acceptance of sugar relationships; however, attachment avoidance moderated the effect of self-esteem on the acceptance of sugar relationships, that is, the negative effect of self-esteem decreased to zero with increasing attachment avoidance. These observations are consistent with certain findings obtained in previous studies. For example, as compared to the low self-esteem associated with anxious attachment, in general (Campbell and Marshall, 2011), the higher levels of self-esteem reported by avoidance attached individuals probably reflect maladaptive narcissism. Smolewska and Dion (2005) found that covert narcissism was better predicted by attachment avoidance than by attachment anxiety.

Furthermore, attachment avoidance moderated the negative effect of intrinsic motivation on the acceptance of sugar relationships, that is, the effect of intrinsic motivation decreased with increasing attachment avoidance. This finding is in line with the observation that intrinsic motivation is an essential part of self-concept of oneself (Ryan and Deci, 2017), which is primarily associated with preference for a sexual relationship based on mutual emotional commitment rather than for sex in return for material compensation (Birkás et al., 2020; Láng et al., 2021); however, intrinsically motivated sexual activity appears to be severely inhibited in those insecurely attached individuals who tend to avoid intimate relationships.

In the older subsample, attachment avoidance moderated the positive effect of extrinsic motivation on the acceptance of sugar relationships, that is, the effect of extrinsic motivation decreased with increasing attachment avoidance. This finding deserves particular attention, given that the observed effect of attachment avoidance is contrary to that found in the younger participants. High extrinsic motivation combined with low attachment avoidance is more likely to be satisfied by attaining social goals (e.g., achieving admiration or respect of others), thus, it is more consistent with engaging in a sugar relationship, in which a young and attractive partner may enhance the impression made by the older partner on their social environment. On the other hand, high attachment avoidance is more likely to be associated with highly egocentric satisfaction of extrinsic motives (e.g., accumulating material assets), which is less consistent with investing in a sugar relationship. These explanatory hypotheses should be tested in future studies possibly utilizing the full version of the AI. Further studies could also profit from using RSES as a two-component measure (e.g., Tafarodi and Milne, 2002; Dessí and Zhao, 2018).

Some limitations of the study must be also acknowledged. Given its correlational, cross-sectional design, causal inferences are hypothetical. Thus, further studies should use longitudinal or follow-up designs to gather data that are more appropriate for making causal inferences. Another limitation of the study is theoretical. Adult attachment theory (Hazan and Shaver, 1987) and the model used in this study (Bartholomew and Horowitz, 1991) could be culturally biased. We deemed adult attachment theory as an appropriate theoretical framework since the sample consisted of individuals living in a Western society with access to the internet; however, these results and discussion should be handled with caution in non-Western societies, given the fact that attachment theory might overclaim universality (e.g., Keller, 2018).

## Data Availability Statement

The raw data supporting the conclusions of this article will be made available by the authors, without undue reservation.

## Ethics Statement

The studies involving human participants were reviewed and approved by Hungarian United Ethical Review Committee. The patients/participants provided their written informed consent to participate in this study.

## Author Contributions

DI: methodology, writing original draft, and funding acquisition. EC: data collection, writing original draft, and funding acquisition. AL: formal analysis and investigation. NM: conceptualization, data collection, review and editing manuscript, and supervision. All authors contributed to the article and approved the submitted version.

## Conflict of Interest

The authors declare that the research was conducted in the absence of any commercial or financial relationships that could be construed as a potential conflict of interest.

## Publisher's Note

All claims expressed in this article are solely those of the authors and do not necessarily represent those of their affiliated organizations, or those of the publisher, the editors and the reviewers. Any product that may be evaluated in this article, or claim that may be made by its manufacturer, is not guaranteed or endorsed by the publisher.
